# Formative evaluation of a participatory women's group intervention to improve reproductive and women's health outcomes in rural Bangladesh: a controlled before and after study

**DOI:** 10.1136/jech-2015-205855

**Published:** 2016-01-06

**Authors:** Helen A Harris-Fry, Kishwar Azad, Leila Younes, Abdul Kuddus, Sanjit Shaha, Tasmin Nahar, Munir Hossen, Anthony Costello, Edward Fottrell

**Affiliations:** 1 UCL Institute for Global Health, University College London, London, UK; 2 Diabetic Association of Bangladesh (BADAS), Dhaka, Bangladesh

**Keywords:** MATERNAL HEALTH, DEVELOPING COUNTR, DIET, FAMILY PLANNING, HEALTH BEHAVIOUR

## Abstract

**Background:**

Women's groups using participatory methods reduced newborn mortality in rural areas of low income countries. Our study assessed a participatory women's group intervention that focused on women's health, nutrition and family planning.

**Methods:**

The study was conducted in three districts in Bangladesh between October 2011 and March 2013, covering a population of around 230 000. On the basis of allocation for the preceding cluster randomised trials, three unions per district were randomly allocated to receive a women's group intervention and three per district were control clusters. Outcomes included unmet need for family planning, morbidity, dietary diversity, night blindness, healthcare decision-making and knowledge of sexual and reproductive health, nutrition and anaemia. A difference-in-difference analysis was used to adjust for secular trends and baseline differences between women taking part in the intervention and a random sample from control clusters.

**Results:**

We interviewed 5355 (91% response rate) women before the intervention and 5128 after (96% response rate). There were significant improvements in women's dietary diversity score (increase of 0.2 (95% CI 0.1 to 0.3)) and participation in healthcare decision-making (proportion increase (95% CI) 14.0% (10.6% to 17.4%)). There were also increases in knowledge about: contraception (4.2% (2.0% to 6.3%)), ways to treat (55.4% (52.2% to 58.5%)) and prevent (71.0% (68.0% to 74.1%)) sexually transmitted infections, nutrition (46.6% (43.6% to 49.6%)) and anaemia prevention (62.8% (60.9% to 64.6%)). There were no significant differences in unmet need for family planning, morbidity or night blindness.

**Conclusions:**

Participatory women's groups have considerable potential to improve women's health knowledge, but evidence of impact on certain outcomes is lacking. Further formative work and intervention development is needed to optimise the impact of this approach for women's health.

## Introduction

Bangladesh has made rapid improvements in women's health over the past few decades, particularly in comparison with other South Asian countries. In 1990, the maternal mortality ratio in Bangladesh was the highest in South Asia at 552 per 100 000 live births, but by 2013 it was the lowest at 243.^1^ It is estimated that 52% of the maternal deaths in Bangladesh that would have occurred in 2010, relative to 2001 rates, were prevented by declines in fertility.^2^ These declines in fertility coincided with the second largest decreases in unmet need for family planning in Asia; unmet need fell by 12.6% between 1990 and 2010.^3^

Despite this progress, the maternal mortality ratio is still higher than the global and developing country averages of 209 and 233, respectively,^1^ and unmet need fell by only two percentage points between 2011 and 2014.^4^ ^5^ With unmet need currently at 12%,^5^ the second target of the fifth Millennium Development Goal, to achieve universal access to reproductive health, was not achieved. Discontinuation and switching between contraceptive methods also remains a problem. Twenty-eight per cent of women using oral contraception stopped due to dissatisfaction, and only half of those who stopped switched to another modern method.^6^

Another related women's health problem in Bangladesh is poor nutrition. Around one-fifth of all Bangladeshi women are undernourished and one-third are anaemic,^4^ ^7^ a problem that is exacerbated for women with high fertility due to prolonged elevated energy, iron and folate needs. Households experience food shortages for around one-quarter of the year,^8^ yet the poor nutritional status of the population is compounded by the ‘double burden’ of both undernutrition and an increasingly overweight and obese population.^9^

The WHO's Bangladesh health strategy recognises that ‘simple interventions’ could improve women's access to health services and their social determinants of health.^10^ One possible intervention is women's groups using ‘participatory learning and action’ (PLA), which was first used by O'Rourke *et al*,^11^ in Bolivia. A meta-analysis of seven cluster randomised controlled trials in Bangladesh, India, Nepal and Malawi found that exposure to women's groups was associated with a 20% reduction in neonatal mortality, and this increased to 33% reduction when at least 30% of the women's group members were pregnant. It also found that women's groups were cost-effective, according to the WHO standards; the cost of women's groups per neonatal year of life lost averted ranged between 91 and 753 international dollars (in 2011).^12^

In Bangladesh, a cluster randomised controlled trial of PLA women's groups in Bogra, Faridpur and Moulavibazar districts reduced neonatal mortality rates by 38%.^13^ A follow-up study in the same areas demonstrated potential to improve a range of child health indicators, including a 15% increase in exclusive breastfeeding, 10% decrease in reported fever and 12% decrease in acute respiratory infections in children under 5 years.^14^ No effect was found for children's dietary diversity or immunisation uptake.

We hypothesised that PLA women's groups in the same communities could also improve indicators of women's and reproductive health in Bangladesh. This paper describes key indicators of women's and reproductive health—including women's dietary diversity score (WDDS), unmet need for family planning and self-reported morbidity—and provides a formative evaluation of a PLA women's group intervention to improve women's and reproductive health.

## Methods

### Study setting and population

The study was implemented by a partnership between the Diabetic Association of Bangladesh (BADAS), Dhaka, Women and Children First (UK), and UCL Institute for Global Health (IGH), London. The three study districts, Moulavibazar, Faridpur and Bogra, were purposively chosen; the districts typify three different topographical features of rural Bangladesh. Moulavibazar district is characterised by its hilly terrain and large tea garden estates. Faridpur district, located in central Bangladesh, contains large rivers which makes some areas difficult to access and causes frequent floods.^15^ The study areas in the northern district of Bogra are comparatively dispersed, and so travel in this district is challenging.

### Intervention

**Figure 1:**
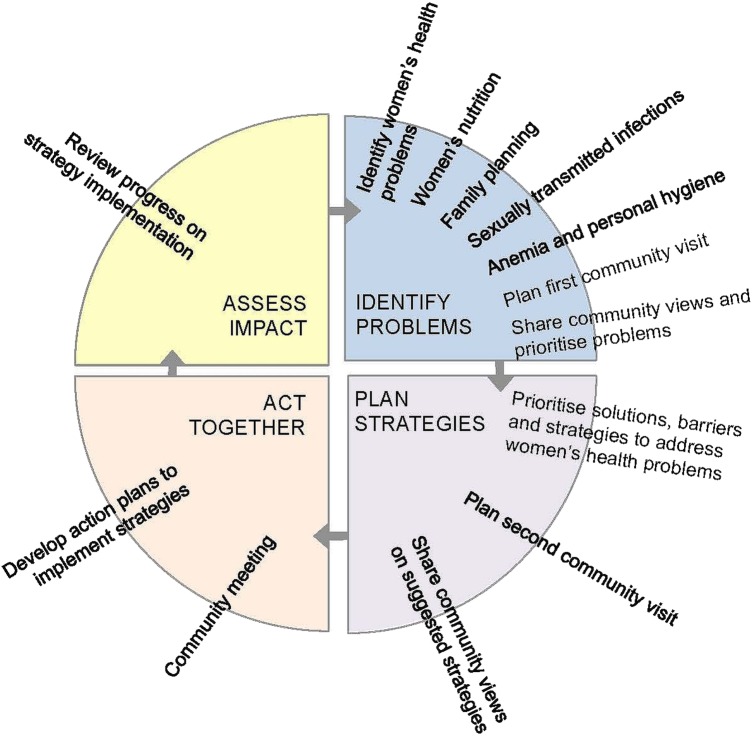
Summary model of the participatory learning and action process and meeting plan.

The women's group intervention, which lasted 13 months, adopted a PLA approach. Women met monthly, in groups facilitated by locally recruited women, to identify problems and implement strategies relating to women's health. This process of prioritising problems, identifying strategies to address these problems and collectively planning, implementing and evaluating these strategies is summarised in figure 1.

**Figure 2:**
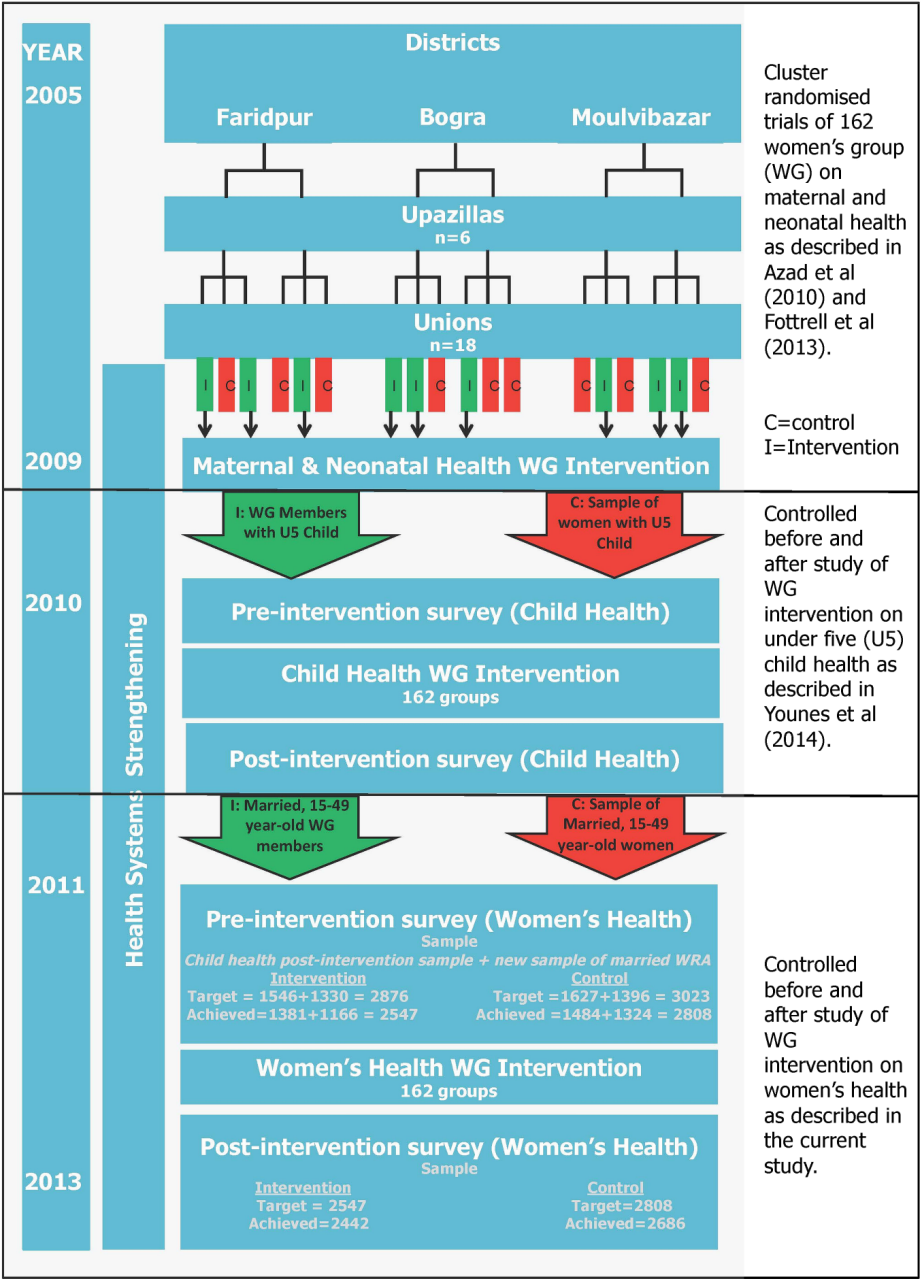
Study process and timeline.

The intervention worked with existing groups that were formed for the earlier interventions, as shown in the timeline in figure 2. The first intervention was a cluster randomised controlled trial that measured the effect of PLA women's groups on maternal and neonatal health, and focused on pregnancy, delivery and postnatal health risks for women and babies.^16^ The second study evaluated the effect of the same groups on child health outcomes, specifically on child nutrition, immunisation and danger signs, common childhood illnesses and accidents and injuries.^14^ The third study, whose results are reported here, measured the effect of PLA women's groups on women's health outcomes. The same groups continued to meet, but women were allowed to join throughout the three studies. In all three studies, the same model of participatory learning and action was applied.

The studies differed in their analyses and samples. The first study was a trial that measured population-level differences between interventions and control clusters, whereas the previous child health PLA and this women's health PLA intervention were formative evaluations to compare differences between women actively participating in the women’s groups, and so highly exposed to the intervention, and a random sample of women from control clusters. These formative evaluations tested different health topics and new materials to inform if the interventions might work on a larger scale, and what modifications might be necessary to achieve population-level, community-wide impacts.

Nine facilitators, recruited for the prior maternal and neonatal health intervention^16^ and child health intervention,^14^ were salaried, trained and retained. They were local married women with at least a high school degree. Similar to the first two interventions, their role was to convene monthly women's group meetings in 162 groups and enable group members to participate in the PLA cycle. Facilitator training lasted 1 week and covered: key health messages on nutrition, sexual and reproductive health, and hygiene (figure 1); methods for group facilitation and fostering participation; and briefing on use of a facilitation manual and flip chart to convey women's health messages during group meetings. Locally recruited supervisors provided support to the facilitators with group meeting preparation and engagement with local stakeholders.

Any woman of reproductive age (15–49 years) who lived in an intervention area was eligible to become a group member. Group membership entailed some commitment to help to organise and take a more active role in group activities. Although groups met on a monthly basis, the intervention was designed to be a continuous process of community mobilisation, with groups being active in between meetings by engaging with communities and planning and implementing strategies. All community members, including men, were welcome to attend and participate in a more passive role and the wider community was engaged throughout the intervention through a series of community meetings (figure 1). These were large public events organised by the women's groups and these events were typically used to discuss key health problems that women identified and prioritised. The women's groups proposed action plans to which the community gave feedback and opinions.

All study sites received health service strengthening to be better equipped to respond to increased community-level demand for health services. These activities focused on technical support and training to frontline health workers, the provision of basic equipment (weighing scales and blood pressure monitors) to community clinics, and facilitation of links between the community, local health planning committees and service providers.

### Intervention allocation

Three unions (clusters) per district (stratum) had previously been randomly allocated to receive the PLA women's group intervention and the remaining three clusters per district had been designated control clusters (figure 2). This gave a total of nine intervention and nine control clusters. Allocation was based on randomisation from the cluster randomised control trial of PLA women's groups on maternal and neonatal health that was conducted between February 2005 and December 2009, and no new randomisation was carried out for the current formative evaluation. The original stratified randomisation process involved the nine union names in a district being written on paper and placed in a bottle, with the first three unions selected from the bottle allocated to the intervention.^16^

### Evaluation

To assess intervention impact, we conducted pre-intervention and post-intervention surveys in October-December 2011 and February-March 2013, respectively. Data were collected through structured face-to-face interviews with women on their socioeconomic status; knowledge, attitudes and practices on family planning; knowledge of symptoms and prevention of sexually transmitted diseases; dietary behaviours; participation in decision-making; and health-seeking behaviours. Interview questions were largely based on the Bangladesh Demographic and Health Survey (BDHS)^4^ and FAO guidelines^17^ to ensure that, where appropriate, results could be comparable. All survey tools were pilot tested before data capture.

On the basis of intracluster correlation coefficients derived from our preliminary and BDHS data, we estimated that a sample of 4800 women at 80% power and with 95% confidence would enable us to detect differences of at least 15% for self-reported morbidity and WDDS, and a difference of 42% for unmet need.

Increasing our target sample by 20% in intervention clusters and 25% in control clusters to allow for non-response, a total of 5899 (2876 intervention; 3023 control) women were included in the pre-intervention survey sample. These women were married women of reproductive age (n=3173 (intervention=1546, control=1627)) who completed a separate child health survey that was administered for the evaluation of a child health intervention. In the intervention clusters, an additional 80% of the remaining married, reproductive-aged women's group members identified from women's group registers (n=1330) were sampled. An equivalent number plus 5% (n=1396) of randomly sampled permanently resident, married women of reproductive age in control clusters were selected from household registers maintained by BADAS (figure 2). All married women of reproductive age included in the pre-intervention survey were eligible for the post-intervention survey.

Thirty-six local data collectors were trained for 3 days on data collection procedures. Approximately 10% of questions of 10% of all interviews were cross-checked through a re-interview by the monitoring coordinator. Hard copies of the completed questionnaires were sent to the surveillance manager at the head office in Dhaka. Further checks for quality and completeness of data took place in Dhaka by the surveillance and data managers. Any omissions identified were referred back to the field for verification. Data were entered into a Microsoft Access database for further checking and cleaning.

### Outcome definitions and analysis

Using USAID and DHS definitions of unmet need for limiting births, women had unmet need if they were at risk of pregnancy, so they were not using any method of family planning, but wanted to wait at least 2 years before their next pregnancy.^4^ ^18^ Women were not at risk of pregnancy if they were unable to have children, were pregnant, were menopausal or had a hysterectomy, were subfecund or infecund, or postpartum amenorrhoeic. WDDS was based on any reported consumption over the previous 24 h of the following nine food groups: starchy staples; legumes and nuts; dairy; organ meat; eggs; flesh meat and fish; vitamin A-rich dark green leafy vegetables; other vitamin A-rich vegetables and fruits; and other fruits and vegetables. Women who reported unusual intakes due to fasting or celebration days within 24 h were excluded from the WDDS analysis, and the score (range 0–9) was the total number of food groups consumed.^17^ Decision-making on healthcare was measured by asking respondents who would take decisions about seeking healthcare if the woman were ill. Women were considered to be involved in decision-making if they reported that they themselves took the decision or if they took the decision jointly with someone else. Prevalence of night blindness was measured by asking respondents if they suffered from night blindness, with a clarification that this meant difficulty seeing in dim light. Ideals (ideal spacing between pregnancies, age at marriage and age at first pregnancy) were measured by asking respondents, in their opinion, what they thought the ideal ages were.

Given that intervention areas were previously exposed to PLA cycles, pre-existing differences in outcome measures or respondent characteristics at baseline were anticipated. We conducted a difference-in-difference regression analysis using a random effects model to account for differences at baseline by measuring the size of the interaction between control-intervention and pre-post intervention. The difference-in-difference estimate is the difference between the pre-intervention and post-intervention mean outcomes in the intervention areas, minus the difference between the pre-intervention and post-intervention mean outcomes in the control areas. The random effects model controlled for clustering within unions and the stratified (three district) nature of the study design. We tested for differences between respondent characteristics for possible inclusion of confounders in a multivariate regression, and used stepwise regression to test for improved goodness-of-fit. Given that we had 18 outcomes, we applied the Bonferroni correction for multiple testing so that results with a p value of 0.003 or less were considered statistically significant at the 0.05 level.

### Ethical approval

Ethical approval was obtained from the ethics committees of BADAS, Dhaka and Institute of Child Health, University College London. Women who chose to participate in the study gave verbal consent and were free to decline or stop the interview at any time.

### Role of funding source

The study was funded by the Big Lottery Fund, UK. The funding body had no involvement in any part of the study design, implementation or analysis.

## Results

### Exposure and coverage of intervention

The total population of the intervention clusters in all three districts was 229 195, so 162 groups provided an average coverage of one group per 1414 population. On the basis of data from the earlier trial, women's group members included 9% of the population of women of reproductive age residing in intervention areas.^16^

Group members were mainly women of reproductive age, their mothers-in-law and adolescents.^16^ Women reported that they attended a mean number of 16 women's groups and community meetings during the intervention period. The groups had on average 19 participants (minimum 13, maximum 28).^14^

The first phase of the PLA intervention focused on participatory learning and problem identification within the groups. Group facilitators used flip charts, games and stories to encourage group discussion of local issues and barriers that women faced to achieving good health. The materials were the same for all groups, but the discussions varied due to the participatory nature of the learning process. At the end of this phase, women prioritised the health issues that they wanted their group to focus on. In the second phase of the PLA cycle, the group activities differed depending on the topic that they had prioritised, as women planned strategies of ways that they could address the problem they had voted on. This involved a number of meetings with communities and community leaders. The third phase was the ‘action’ phase where the groups implemented their strategies. Though strategies inevitably varied between groups, common strategies included raising awareness of the messages in the PLA flip chart using methods such as community meetings, door-to-door visits and dramas. Another common strategy was emergency group funds that carried on from the previous cycles on maternal, newborn and child health and were used to enable transport and access to healthcare. Informal evaluation of these strategies by the groups informed further action, representing phase four of the PLA cycle.

### Response rates

A total of 2547 (89%) of a target of 2876 women in intervention areas and 2808 (93%) of 3023 women in control areas were interviewed in the pre-intervention survey. A total of 2442 (96%) and 2686 (96%) of the same women were re-interviewed in the post-intervention survey in intervention and control areas, respectively, giving overall response rates of 91% and 96% for the pre-intervention and post-intervention surveys. Reasons for failure to interview included: migration out of the study area, inability to locate the respondent, and the respondent not meeting age inclusion criteria. The 227 non-responders did not differ from responders in terms of religion, asset ownership, pregnancy status, literacy or intervention allocation (intervention or control arm), but non-responders in control clusters were significantly younger than responders in the same arm (29 years vs 31.1 years, t=−2.85, p=0.004).

For question-specific response rates, 6.7% and 12.6% respondents reported that they fasted or had a celebratory feast day in the pre-intervention and post-intervention surveys, respectively, and those respondents were excluded from the analysis of intervention effect on dietary diversity. Those who fasted or feasted 24 h before the post-intervention survey had slightly but significantly more assets (7.5 compared with 7.3 assets; p=0.0188) and more Hindus fasted or feasted than Muslims (χ^2^=9.7708; p=0.021). Of the 20.3% and 10.4% of respondents who reported any illness in the pre-intervention and post-intervention surveys, all of them answered about whether they sought care. All other outcomes had high response rates (89% or higher).

### Study population characteristics

The baseline respondent characteristics in control and intervention are shown in table 1. Respondents had a mean age of over 30 years at the pre-intervention survey (mean 31 years; range 15 to 49) and almost 90% of respondents were Muslim; all remaining respondents in the presurvey were Hindu. Nearly two-thirds of respondents could read easily or with difficulty. Respondents reported ownership of around seven assets per household of an option of 22 assets, such as having electricity, a fridge or a table. t Tests and χ^2^ tests (table 1) found that the control sample were significantly younger, had a higher proportion of Muslims, owned more assets and were more literate than intervention areas. Adjustment for these baseline differences by including the age, literacy, asset ownership and religion in the random effects regression model had a negligible effect on difference-in-difference estimates or CIs (results not shown). Since the differences were not considered to have introduced confounding in the assessment of the intervention effect on study outcomes, the adjustments were not included in the models.

**Table 1:** Summary of respondent characteristics at the pre-intervention survey

Characteristic	Control n=2808	Intervention n=2547	Baseline differences	
			Test statistic	p Value
Age (%)				
Mean (years)	31.0	31.7	t(5353)=−3.18	0.002
≤19	4.7	2.8		
20–24	18.4	17.5		
25–29	24.4	24.5		
30–34	19.8	20.1		
≥35	32.7	35.1		
Religion (%)				
Islam	89.0	87.1	χ^2^=4.84	0.028
Pregnancy status (%)*				
Pregnant	5.3	5.4*	χ^2^=0.04	0.851
Assets				
Mean number of assets owned out of a list of 22 items	6.9	6.6	*t*(5353)=3.89	<0.001
Literacy				
Can read (easily or with difficulty)	64.5	58.0	χ^2^=23.30	<0.001

*Two records with missing information on this variable.

### Intervention impact

On the basis of the Bonferroni corrected p values, there was no evidence of intervention impact on outcomes relating to sexual health or morbidity, but there were minor significant increases in dietary diversity and large improvements in certain knowledge-based outcomes (table 2). Respondents’ knowledge of types, ways to prevent and ways to treat sexually transmitted infections increased in intervention areas compared to control areas by 16%, 71% and 55%, respectively. A significant increase in knowledge of modern contraception methods was also observed in intervention areas, but there was no change in the use of modern contraceptives. The proportions of women who could name ways to prevent undernutrition and anaemia and reported a participatory role in healthcare decision-making also increased significantly in intervention areas. A small but significant reduction in the reported ideal number of years between pregnancies was observed in intervention areas.

**Table 2:** Cluster mean difference-in-difference results of the impact of women's group intervention on women's health indicators

	Control		Intervention		DID* estimate (95% CI)	p Value
	Pre	Post	Pre	Post		
Sexual health						
Unmet need for spacing (%)	5.5	4.8	5.2	3.8	−0.7 (−2.5 to 1.0)	0.391
Unmet need for limiting (%)	21.6	20.6	22.5	17.6	−3.9 (−7.1 to −0.7)	0.018
Total unmet need (%)	27.1	25.3	27.7	21.4	−4.6 (−8.0 to −1.2)	0.009
Knowledge of ≥3 methods of modern contraception (%)	86.5	87.7	92.9	98.3	4.2 (2.0 to 6.3)	<0.001†
Women accessing modern contraception (%)	51.0	52.1	54.1	57.5	2.4 (−1.3 to 6.1)	0.209
Awareness of ≥3 STIs (%)	0.8	0.7	<0.0	15.8	16.0 (14.7 to 17.2)	<0.001†
Awareness of ≥1 way to prevent STIs (%)	36.2	43.5	18.4	96.8	71.0 (68.0 to 74.1)	<0.001†
Awareness of ≥1 way to treat STIs (%)	34.3	51.8	24.5	97.4	55.4 (52.2 to 58.5)	<0.001†
Reported ideal age for marriage (years)	18.5	18.6	18.4	18.3	−0.1 (−0.3 to <−0.1)	0.014
Reported ideal age for first pregnancy (years)	20.9	21.0	20.7	20.7	−0.1 (−0.3 to <0.1)	0.069
Mean ideal spacing between pregnancies (years)	4.6	4.5	4.7	4.0	−0.6 (−0.7 to −0.5)	<0.001†
Nutritional health						
Women's dietary diversity score (mean)	3.9	4.1	4.0	4.3	0.2 (0.1 to 0.3)	0.002†
Night blindness (%)	12.3	12.5	6.3	9.6	3.0 (0.8 to 5.2)	0.008
Knowledge of ≥3 ways to maintain good nutrition (%)	27.0	29.3	45.6	94.5	46.6 (43.6 to 49.6)	<0.001†
Knowledge of ≥3 ways to prevent anaemia (%)	1.8	2.2	3.2	66.2	62.8 (60.9 to 64.6)	<0.001†
Morbidity						
Any illness or injury over the previous 3 months (%)	21.3	10.0	19.4	11.0	3.0 (0.2 to 5.7)	0.033
Sought care for mild or severe self-reported illness (%)	92.1	93.2	92.0	93.4	0.2 (−5.2 to 5.6)	0.938
Women's participation in healthcare decision-making (%)	44.7	33.5	55.6	58.3	14.0 (10.6 to 17.4)	<0.001†

Response rates for all outcomes were 89% or higher, apart from care seeking, which was 20.3% and 10.4% for pre-intervention and post-intervention surveys, respectively, because it was only reported if the respondent had been ill in the previous 3 months. Also, dietary diversity score response rates were 93.3% and 87.4% because scores were not recorded if the respondent had fasted or feasted in the 24 h before the survey.

*Difference-in-difference derived from a random effects model interaction term between pre/postsurvey and control/intervention arm and accounting for the stratified, clustered study design.

†Significant at the 0.05 level using the Bonferroni correction for multiple testing (ie, p value of 0.003).

STIs, sexually transmitted infections.

## Discussion

Our formative evaluation shows that participation in women's and reproductive health PLA women's groups improved women's dietary diversity and knowledge about sexual health, morbidity and nutrition. In particular, our findings show large, positive and significant improvements in women's knowledge of ways to prevent and treat sexually transmitted infections and good nutrition and anaemia prevention. Improvements in women's dietary diversity were small but significant. While there are indications of improvements in outcomes relating to unmet need for family planning and self-reported morbidity, the observed changes failed to reach statistical significance when corrected using the Bonferroni adjustment. As a proof-of-principle assessment of the potential of women's groups to address women's and reproductive health, our findings apply to women who participated in the intervention, but the mixed results suggest a need for further intervention refinement before population-level evaluation.

The study strengths include the very high response rate (over 90% for all surveys), perhaps due to the experience of the data collectors and the use of Bonferroni correction of p values for multiple outcome testing which, as a conservative method, gives us confidence in the effects that we have reported as significant.^19^

Although WDDS indicates the nutritional adequacy of women's diets in Bangladesh,^20^ ^21^ there are no guideline cut-offs to distinguish adequate from inadequate diets.^22^ A new score published after the study proposed 5 of 10 different food groups as ‘adequate’,^23^ although we were unable to calculate this using food groups listed in the survey. It is difficult to know if an increase of 0.2 in WDDS has meaningful implications for nutritional adequacy, but it has been shown that WDDS is moderately correlated with the mean probability of adequate intake for a number of micronutrients.^20^ This suggests that WDDS works well as an indicator of nutritional adequacy, and a small increase might have meaningful nutritional implications.^20^ Similarly, it is difficult to quantify the practical meaning of the small change in knowledge of contraception. However, the BDHS found that only 0.3% of women not intending to use family planning gave lack of knowledge of family planning methods as their reason, so we expect that the implications for this small change in knowledge may be limited.^4^ Broadly, it appears that the PLA women's group intervention was more effective in changing health-promoting knowledge than practice. This may be due to a time lag; any changes in attitudes and behaviours may take longer to come into effect than changes in knowledge. Alternatively, given the breadth of topics discussed, a more specific intervention that focused solely on diet or family planning, for instance, may have resulted in a greater impact on either outcome.

This study was conducted with well-established women's groups and with experienced facilitators; some facilitators had practised the PLA approach for 7 years. This means that, although we have been able to account for differences in baseline between intervention and control areas, the intervention areas are familiar with the PLA approach and communities may have had a greater propensity to respond positively to the intervention. Therefore, the impacts observed might have been quicker to facilitate than in virgin sites. Alternatively, the outcomes may have been limited by the focus on child health from the earlier interventions. Many women's groups continued strategies that were implemented for the child health cycle, such as breastfeeding and emergency funds.^14^ While these strategies are relevant to women's health, groups in fresh sites may have implemented new strategies that are closer determinants of the study outcomes.

The limited change in health practices may also be due to the sociocultural context of rural Bangladesh, in which women may face limited agency to act on new knowledge.^24^ ^25^ If so, the improvement in women's participation in healthcare decisions would be a positive step towards effecting behaviour change. Nevertheless, health is influenced by numerous economic, social, structural and environmental factors, such as poverty, access to land, education, cultural norms, infrastructure and environmental change.^8^ ^26^ Demand-side behaviour changes alone may be insufficient to change the health outcomes being addressed, and so future studies could test the intervention with a bigger health system strengthening component and increase linkages between women's groups and community health clinics. Alternatively, future work could take a longer term approach to explore the scope for women's groups to change these wider determinants of health, and to measure the effect of interactions between women's groups and these wider contextual factors on women's health outcomes. Qualitative research is also needed to characterise context-specific barriers within behaviour change pathways if future PLA interventions wish to maximise their impact.

## Conclusion

Direct participation in a PLA women's group intervention resulted in large and significant increases in women's health knowledge relating to family planning and nutrition, as well as in decision-making relating to accessing healthcare. Qualitative work is required to investigate how the intervention could be adapted to translate these increases in knowledge into improvements in health outcomes that may in turn be observed at the population level, beyond those directly engaged with the intervention.

**What is already known on this subject**

- Women's groups using participatory learning and action approaches have been shown to be cost-effective in improving a range of health outcomes, including neonatal mortality, maternal mortality and child health.^12–14^ ^16^
- The potential for this approach to improve knowledge, attitudes and practices relating to women's health remains to be tested.

**What this study adds**

- Women's groups using a participatory learning and action approach in rural Bangladesh significantly improved women's dietary diversity and their health knowledge, such as ways to maintain a healthy diet and treat sexually transmitted infections. Participation in decision-making related to healthcare seeking was also significantly improved.
- Changes in some behaviours (increased uptake of family planning and self-reported morbidity) were limited and not significantly different, perhaps due to a time lag between acquiring knowledge and adopting new habits.
- Future studies to test a more focused intervention in virgin sites, with qualitative work to identify barriers to a quick uptake of new behaviours, are required before scale-up and assessment at the population level.
